# Comparing paper-based and mobile application for rank-based peer assessment in interprofessional education: before, during, and after the COVID-19 pandemic

**DOI:** 10.1186/s12909-024-06382-2

**Published:** 2024-11-27

**Authors:** Doni Widyandana, Prattama Santoso Utomo, Ide Pustaka Setiawan, Yustina Tyas Kurniawati, Sucheta Dandekar

**Affiliations:** 1 Department of Medical Education and Bioethics, Faculty of Medicine, Public Health and Nursing, Universitas Gadjah Mada, Yogyakarta, Indonesia; 2 Community and Family Health Care – Interprofessional Education Unit, Faculty of Medicine, Public Health and Nursing, Universitas Gadjah Mada, Yogyakarta, Indonesia; 3 Era’s Lucknow Medical College, Lucknow, India

**Keywords:** Interprofessional education, Peer assessment, Mobile application, COVID-19 pandemic era

## Abstract

**Background:**

Education was affected during the COVID-19 pandemic, and there was a need to adapt the learning approaches to the situation. At the University of Gadjah Mada, many essential soft skills of healthcare professionals are taught using the interprofessional education (IPE) approach on-site. Our university responded to this crisis by offering online classes and similar types of training. Post-administration of the course, a peer-assessment was conducted, and it was used to provide feedback on the work or performance of peers among students. Peer assessment was done using paper-based and a mobile application during COVID-19. This study aimed to share a best practice for the implementation of a rank-based peer-assessment application for longitudinal interprofessional education in the community setting and to compare the score distribution of a rank-based peer-assessment before and after using mobile application.

**Methods:**

Quantitative research design was used by processing secondary data on student peer assessment scores from 3 bachelor programs (medicine, nursing, nutritionist) Community and Family Health Care with Interprofessional Education (CFHC-IPE) in Faculty of Medicine, Nursing, and Public Health, Universitas Gadjah Mada. 4,790 students from 2018 to 2022 (5 years) were tracked before, during, and after the COVID-19 pandemic. The assessments carried out were offline, online, and blended learning, respectively. The assessment was carried out using a paper-based method before and during the pandemic, online-based with a mobile application was used. Thus, the peer-assessment was conducted manually or using paper-based method using a 5-rank scoring system. In 2019, the peer assessment was carried out using a mobile application and applied a 10-rank scoring system.

**Results:**

The rank-based peer assessment can be well implemented to make students assess their friends more objectively, with an average score 82.02 ± 8.68. The rightward shift in the distribution of scores indicates that the average score has improved after using the mobile application compared to before its use. (82.02 ± 8.68 vs. 62.39 ± 11.13, *p* < 0.05).

**Conclusion:**

The implementation of rank-based peer-assessment using a mobile application was well received during and after the pandemic by interprofessional undergraduate students. The online system made the assessment more objective and the average grades were seen to be better.

**Supplementary Information:**

The online version contains supplementary material available at 10.1186/s12909-024-06382-2.

## Background

COVID-19 pandemic provided us with an experience of multidimensional disturbance. Education was also affected as there was a need to adapt the learning approaches using online learning. Online learning gained momentum during the COVID-19 pandemic. This included medical and health professions education. Online learning proved to be effective in achieving learning outcomes in medical education [1], despite its drawback for integrating clinical learning [2]. After the pandemic, the use of online learning continues to be maintained in the learning activities, particularly the use of online lectures and asynchronous learning. Online/distance education has gained favor as a learning measure as it is more accessible and effective [3, 4].

Interprofessional education is a learning approach to promote students’ experience and prepare the students for their future clinical practice. The interprofessional approach is related to interprofessional practice and patient-centered care [5] and the sessions are delivered in many learning models based on the intended outcomes, from lectures, small-groups, clinical simulations, mentoring, and community observations [6–9]. The use of small-group learning is preferred in interprofessional education to ensure a more in-depth discussion and immersion with the multi-professional team members. Evidence shows that the use of small-group learning in interprofessional education can be best used to nurture collaborative performance among group members [10, 11].

Though the teaching-learning activities have got streamlined, there has been a challenge in assessing interprofessional practice and education of health professions. As interprofessional learning activities are usually conducted in small groups, the contact time and activities are mostly student-led activities. In this context, providing students with the opportunity for peer assessment can be highly beneficial. Peer assessment involves students evaluating and providing feedback on the work or performance of their peers. By incorporating peer assessment into the assessment process, several advantages are realized, particularly when direct observations are involved [12]. Studies reported that the use of peer assessment can be considered as a valid assessment item if the assessors are provided with sufficient grading skills and training [13]. Peer assessment might supplement other assessments to achieve a 360-degree assessment goal [14].

One of the threats of peer assessment is the phenomenon of failure to fail, where students from the same group might be inclined to rate other students with the highest score possible and the same scores. Moreover, they might hesitate to provide lower scores to their peers as they are also afraid to receive scores from others [15]. The phenomenon might occur due to the dilemma of emotions attached among friends [16]. Hence, there is a need to create an assessment system to facilitate objective peer assessment. Rank-based assessment is an assessment undertaken based on the performance of students in a group, where students will be scored differently based on their rank of participation and performance [17]. Hence, it will encourage graders to provide clear demarcations between students’ individual performance fairly and objectively, from most active to passive. In this context, peer rank-based assessment may prevent students from providing the same scores as it is using an application system. The scoring system will maintain objectivity but not impose lower scores for students.

Our institution implemented a rank-based peer-assessment application in interprofessional education to encounter the challenge and to ensure an objective and safe peer assessment. The peer-assessment used scales for rank-based assessment, initially based on 5-rank scores and then changed to 10-rank scores. The 10-rank scores would result in a more valid assessment, thus providing distinctive scores and having higher reliability [18].

This study aims to share a best practice for the implementation of a rank-based peer-assessment application for longitudinal interprofessional education in the community setting. A mobile application was then introduced, and the study further compared the score distribution of a rank-based peer assessment before and after using the mobile application.

## Methods

### Context and setting

The study explored assessment used at a community-based interprofessional education program called Community and Family Health Care – Interprofessional Education (CFHC-IPE). It is a flagship program of the Faculty of Medicine, Public Health and Nursing, Universitas Gadjah Mada, Yogyakarta, Indonesia. CFHC-IPE is a longitudinal program which runs through the 3.5 years (7 semesters) of the undergraduate curricula of the medicine, nursing and health nutrition programs. The program is on-going and has been established since 2019. The program curriculum encourages students to learn together, to apply science and skills that they already have garnered from the class to the families in the community around the faculty. The program serves 2,610 families in Sleman, municipality surrounding the university.

The CFHC-IPE program’s assessment blueprint applies a 360-degree assessment system, to assess students based on the point of views of lecturers, supervisors, community advisors, community/family members and also peers/fellow students. Initially, the peer assessment involved a paper-based open-scoring system. The peer assessment results, however, showed that students provide best/highest possible scores to all group members despite their varying performance. In 2017, the community-based interprofessional education program started to apply a rank-based peer assessment as one of the assessment methods to evaluate students’ achievement on the program’s learning objectives. The peer assessment is conducted after each semester. The scoring aspects include attendance, responsibility, interprofessional communication, collaboration, contribution and respect, and also community engagement. The peer-assessment was conducted manually using a 5-rank scoring system. In 2019, a mobile application was developed and this used a 10-rank scoring system. With the mobile application, students have the opportunity to assess and provide feedback on their peers’ work. However, to ensure fairness and avoid inflated assessments, certain regulations are in place. These regulations discourage the tendency to assign consistently high scores to all students. The application also provides room for constructive feedback and scores from the supervisors and advisors. Hence, the assessment processes are thoroughly documented and ease the work of the administrators. The program was conducted in-person before the pandemic, but changed to fully online during the pandemic. As the pandemic eased, the program is delivered as a hybrid or blended learning approach.

A retrospective quantitative approach cohort study was conducted to explore students’ peer assessment scores and experience before and after the implementation of the mobile application. The study evaluated student peer-assessment score differences and pattern changes before and after the implementation of the mobile application.

### Participants

A total of 4,790 students participated in this evaluation program from 3 undergraduate programs (i.e., medicine, nursing, and health nutritional sciences) from batch/year 2017 to 2021. Consent was obtained from the participants. The study received ethical clearance from the Human Research Ethics Committee of the Faculty of Medicine, Public Health and Nursing, Universitas Gadjah Mada, (Ref No. KE/FK/0970/EC/2022).

### Data collection

The main instrument for data collection was the mobile application for peer assessment. The data collection was done after the last feedback session by the faculty advisor after the field trip of students in the family or community in each semester. The mobile application is a web-based application developed, which can be accessed at https://app.cfhcipeugm.id/. The application allows easier data collection, and the students can perform peer assessment more independently. Earlier, a manual method was used within the team at one time and was not supervised by the faculty advisor, resulting in a more subjective assessment. The manual method (referred to as the paper-based method) is also prone to errors and data loss. The assesment scores are longitudinal across semesters with the range of 0 to 100. The mobile application, developed at Yogyakarta by the CFHC-IPE program in collaboration with CareNusa team, allows peer-to-peer assessments to evaluate peer’s performance within the team throughout the semester of the program.

**Fig. 1:**
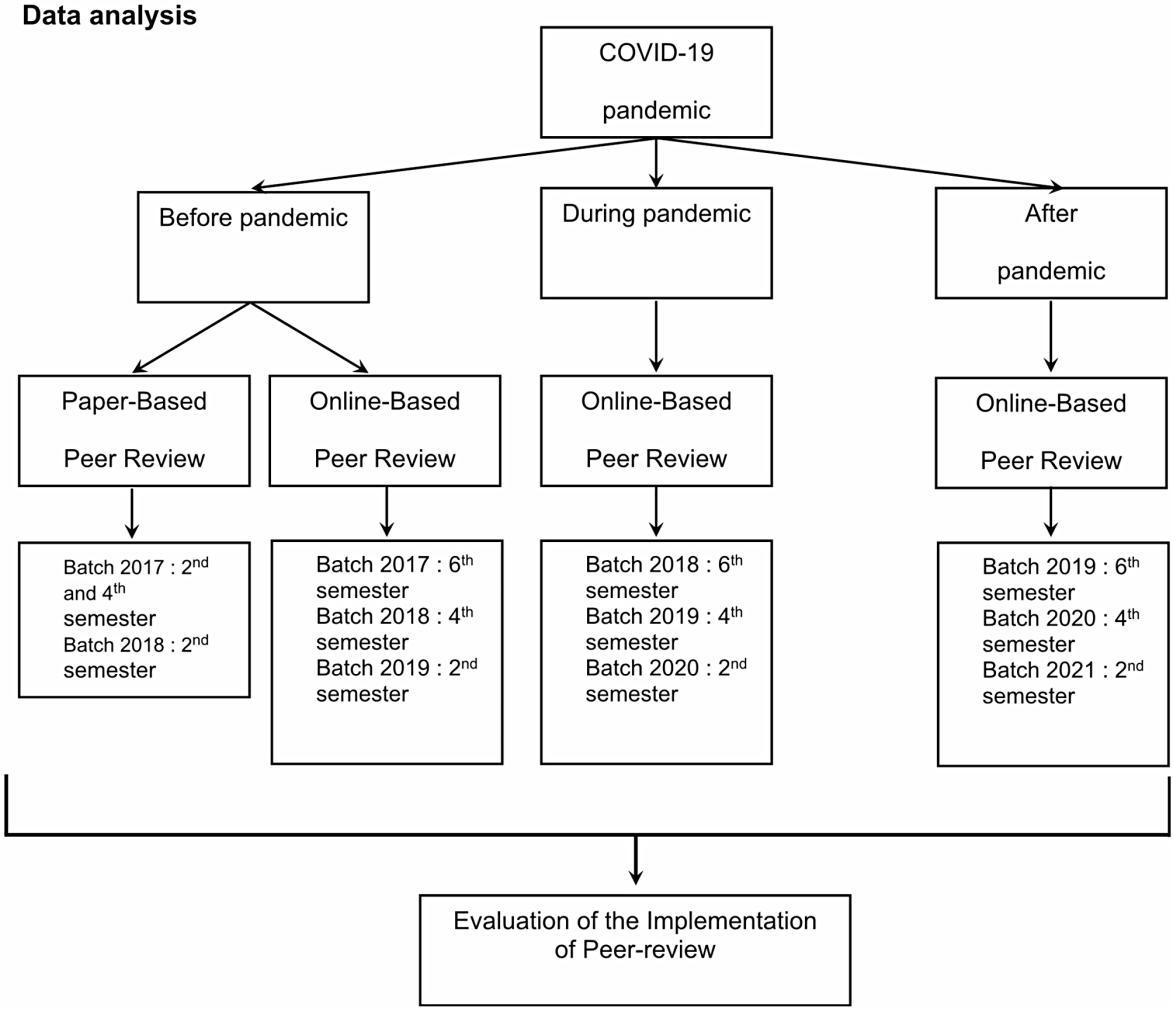
Scheme of sample analyzed

Quantitative data were the peer assessment scores before and after the mobile application implementation; taken before, during pandemic, and the post-pandemic periods, details provided in Figure 1.

The peer assessment scores were analyzed using descriptive analysis and compared based on the timeline (i.e., before and after implementation of the mobile application) and based on the learning environment (i.e., before, during the COVID-19 pandemic, and post-pandemic period). Peer-assessment which are analyzed from 2017 to 2021 academic year (2nd, 4th, and 6th semester). Before pandemic, peer assessment which analyzed are 2nd, 4th, and 6th semester of 2017 academic year also 2nd, 4th semester of 2018 academic year and 2nd semester of the pandemic, peer assessments were analyzed in the 2nd, 4th, and 6th semesters of the 2017 academic year, the 2nd and 4th semesters of the 2018 academic year, and the 2nd semester of the 2019 academic year. During pandemic, peer-assessment which analyzed are 6th semester of 2018 academic year also 4th semester of 2019 academic year and 2nd semester of 2020 academic year. Post academic, peer assessment which analyzed are 6th semester of 2019 academic year also 4th semester of 2020 of 2020 academic year and 2nd semester of 2021 academic year. The comparative analysis was based on bivariate analysis (t-test) and multivariate analysis using ANOVA and Tukey’s multiple comparison or Post-Hoc Test.

## Results

The total sampling method implemented in this research including a total of 4,790 students participated in this evaluation program from 3 undergraduate programs (i.e., medicine, nursing, and health nutritional sciences) from batch/year 2017 to 2021. The participants/students were grouped in groups consisting of 4–5 students from 3 undergraduate programs. Each group was guided by a faculty advisor and a field instructor. Participants characteristics in details are provided in Table 1. There were 3,699 female students (77.2%) and 1,091 (22.8%) were male students, which consisted of 2,727 (56.9%) from medicine study program, 954 (19.9%) from nursing study program and 1,109 (23.2%) from health nutrition study program. The faculty advisors were from 3 undergraduate study programs: medicine, nursing and health nutrition who guided students in the five academic years. The faculty advisors were from the following background as following: 2,637 (55.1%) medical doctors, 1,002 (20.9%) nurses, 548 (11.4%) health nutritionists, 224 (4.7%) public health, and 379 (7.9%) allied health. Besides that, this activity also involves field instructors.

Each interprofessional group will be accompanied by one faculty advisor and field instructor too. Field instructors are recruited from community health center officers in the Sleman regency with diverse professional backgrounds (i.e.: medical doctor, midwife, nurse, health nutritionist, or public health) and trained by Community and Family Health Care with Interprofessional Education (CFHC-IPE) to be able to accompany groups in the field. Their tasks are guiding students both online and offline, helping to connect with partner families, guiding them for needs assessments and preparing appropriate programs for families and communities according to predetermined placements. They will also play a role in providing assessments to students together with field supervisors from campus. In addition, field instructors were involved from different professions, for instance, 1,627 (34.0%) medical doctor, 752 (15.7%) midwife, 1,407 (29.4%) nurse, 827 (17.3%) health nutritionist, and 176 (3.7%) public health.

There were two kinds of peer assessment methods which consisted of manual methods which were done by 1,073 (22,4%) students and mobile methods which were done by 3,717 (77.6%) students. These peer assessments were done within three periods of time: before pandemic periods, during pandemic periods, and post pandemic periods which were done by 1,907 (39.8%), 1,596 (33.3%) and 1,287 (26.9%) students respectively. Delivery of learning was by in-person/offline, online and blended following those three periods of time. This activity involves 13 sub districts in Sleman regency, Yogyakarta province.

**Table 1:** Characteristics of participants

Variables	Total *N* = 4,790 *n* (%)
**Gender**	
Female	3,699 (77.2%)
Male	1,091 (22.8%)
**Study Program of Students**	
Medicine	2,727 (56.9%)
Nursing	954 (19.9%)
Nutrition	1,109 (23.2%)
**Academic Year of Students**	
2017	975 (20.4%)
2018	1,269 (26.5%)
2019	1,233 (25.7%)
2020	874 (18.2%)
2021	439 (9.2%)
**Profession of Faculty Advisor**	
Medical Doctor	2,637 (55.1%)
Nurse	1,002 (20.9%)
Health Nutritionist	548 (11.4%)
Public Health	224 (4.7%)
Allied Health	379 (7.9%)
**Profession of Field Instructor**	
Medical Doctor	1,627 (34.0%)
Nurse	1,407 (29.4%)
Nutritionist	827 (17.3%)
Midwife	752 (15.7%)
Public Health	176 (3.7%)
**Media of Peer Assessment**	
Manual	1,073 (22.4%)
Mobile Apps	3,717 (77.6%)
**Period of Peer Assessment**	
Before Pandemic	1,907 (39.8%)
During Pandemic	1,596 (33.3%)
Post Pandemic	1,287 (26.9%)
**Delivery Method of Learning**	
Offline	1,907 (39.8%)
Online	1,596 (33.3%)
Blended	1,287 (26.9%)

**Fig. 2:**
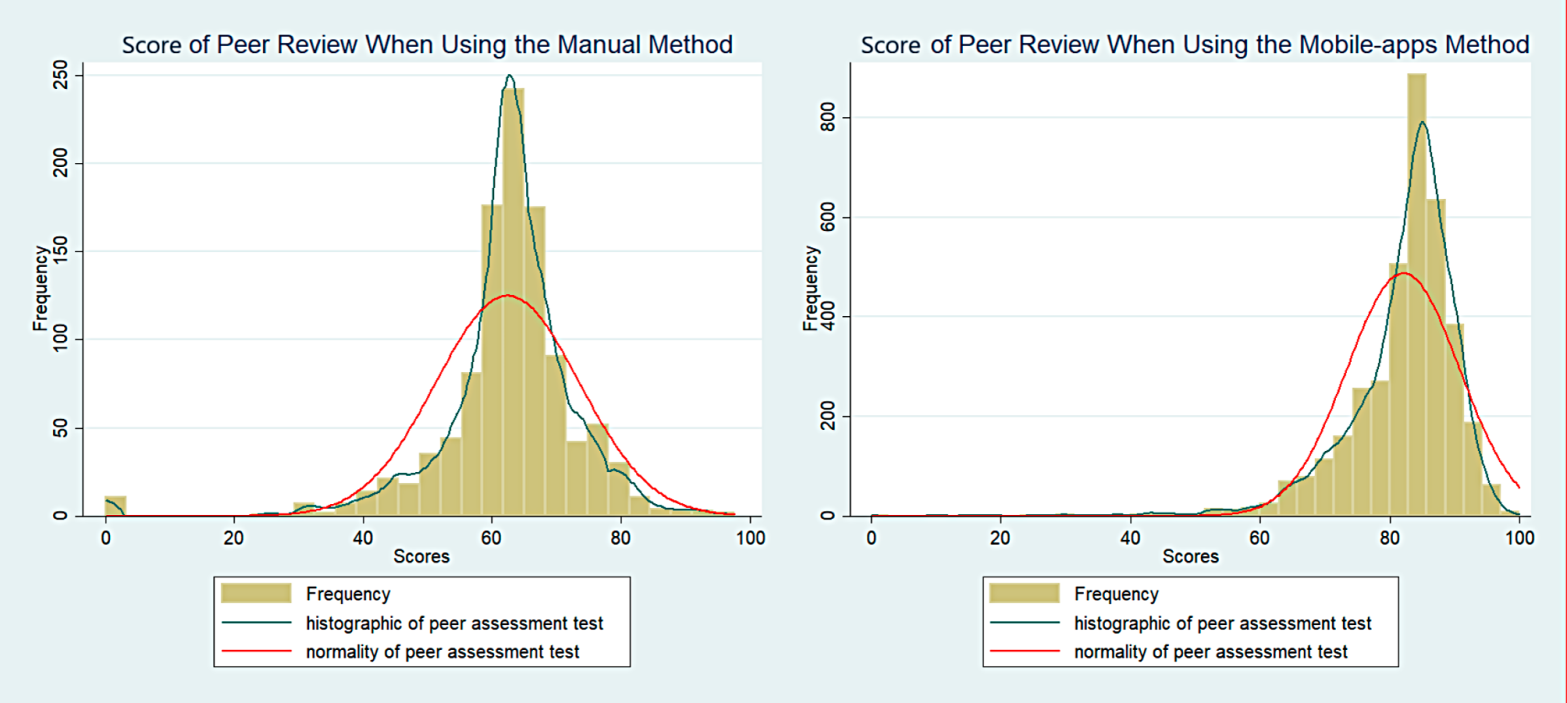
Graph of Peer Review Score Based on Methods

Figure 2 below shows the result of rank-based peer assessment was normally distributed. Peer review assessment using webapps methods showed the curve shifts to the right. The mean and median of rank-based peer assessment using webapps method was higher than peer assessment using manual method.

The differences between manual and mobile apps method scores of peer review are identified. The mean score of the mobile apps method (82.02 ± 8.68) is higher than the mean score of the manual method (62.39 ± 11.13). And both mean scores are statistically different due to the p-value shown < (0.05) which means any significant differences between the two variables (Table 2).

**Table 2:** Differences between manual and mobile apps method scores of peer review

Media of Peer Assessment	Mean ± SD	95% CI	*p*-value*
Manual	62.39 ± 11.13	61.73–63.06	0.000
Mobile apps	82.02 ± 8.68	81.74–82.30	

*t-test p-value

**Fig. 3:**
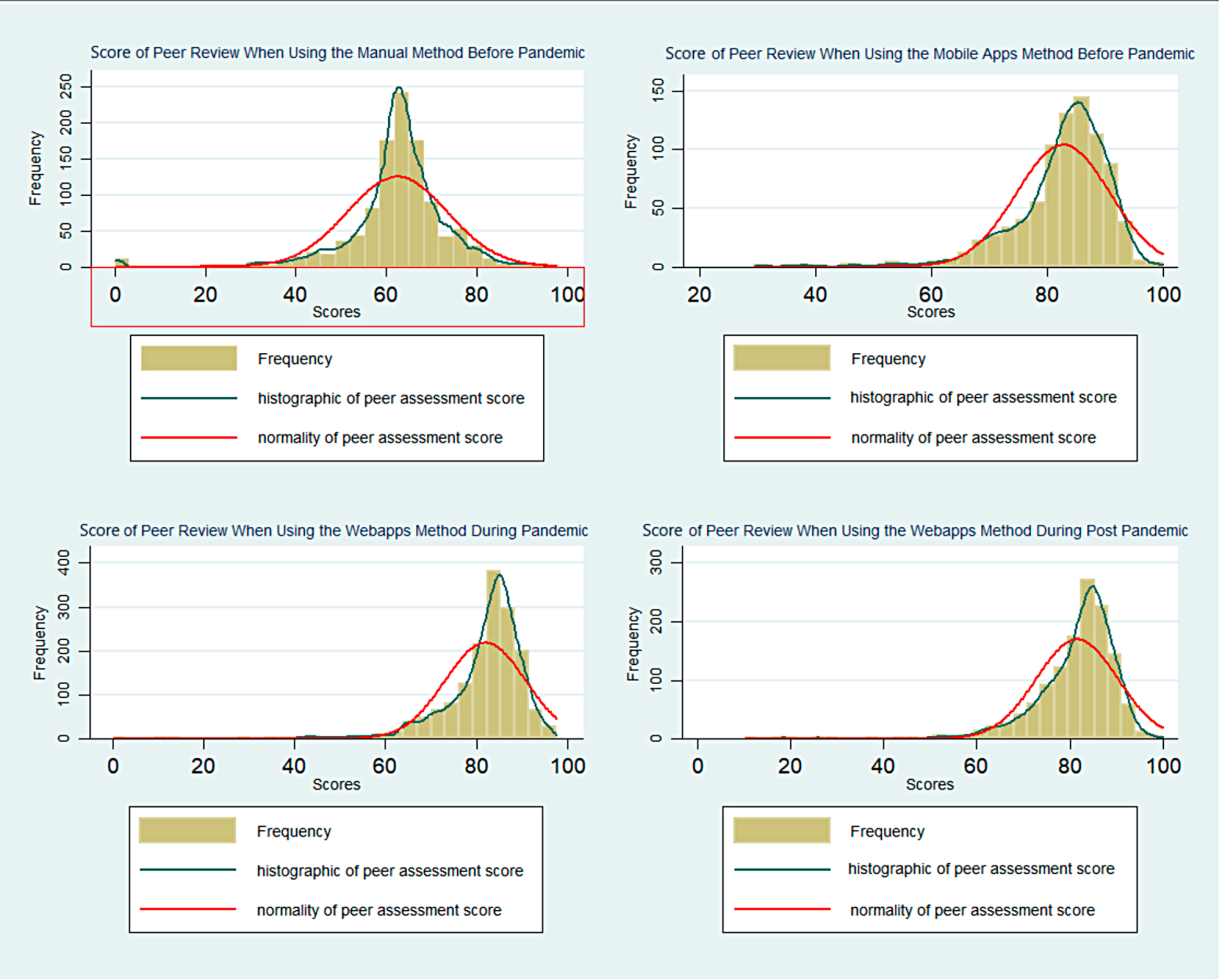
Peer assessment score distribution among different time periods

Furthermore, participants were classified into three groups based on the periods of peer assessment, for instance, a group with peer assessment scores before the pandemic (*n* = 1,925), during the COVID-19 pandemic (*n* = 1,618), and post-COVID-19 pandemic (*n* = 1,309). Comparison graph of peer assessment scores based on those three groups are illustrated in Fig. 3.

We then conducted a one-way ANOVA test to determine if the peer review scores would differ among the periods of the peer review assessment. The data shown is in the form of mean ± standard error. The ANOVA test found that the results differed statistically significantly between the three groups (p-value < 0.05 (*p* = 0.000)). The comparison of mean scores in the three periods showed the scores in the pandemic periods were the highest and the scores in the pre-pandemic period were the lowest (Table 3).

**Table 3:** Comparison of Mean scores among three periods

Periods	Mean ± SE	*p*-value
Pre-pandemic	71.33 ± 0.32	0.000*
During pandemic	81.96 ± 0.22	
Post-pandemic	81.58 ± 0.24	

*ANOVA p-value

The post-hoc Tukey test is done to determine exactly which scores are significantly different. The test showed that the peer review score was the highest during pandemic compared to pre-pandemic (10.63 ± 0.38 points, *p* = 0.000) and during post-pandemic compared to pre-pandemic (10.25 ± 0.41 points, *p* = 0.000). However, the differences were not found statistically in peer review scores during post-pandemic compared to pandemic (-0.37 ± 0.42 points, *p* > 0.05) (Table 4).

**Table 4:** Post-hoc comparison of Mean scores between two periods

Differences	Mean ± SE	*p*-value	
Pandemic vs. Pre Pandemic	10.63 ± 0.38	0.000*	
Post Pandemic vs. Pre Pandemic	10.25 ± 0.41	0.000*	
Post Pandemic vs. Pandemic	-0.37 ± 0.42	0.647	

*p < 0.05, statistically significant

## Discussion

This study highlights that rank-based peer assessment can be well implemented in an interprofessional education involving 3 health professions programs (medicine, nursing, health nutrition) both before, during, and after the pandemic. Interprofessional education (IPE) is a crucial starting point for collaboration in healthcare practice. This approach presents an opportunity for professionals from different fields to come together, interact, and learn from one another. By doing so, they can gain an understanding of each other’s roles, responsibilities, and expertise, which leads to better collaboration and ultimately enhances the quality of patient care.

IPE encourages a collaborative culture by promoting open communication, mutual respect, and shared decision-making. It provides a platform for healthcare providers to engage in meaningful discussions and develop a common language, which ultimately leads to a more cohesive and effective healthcare team [8].

This study showed rank-based peer assessment implementation did not skew the score distribution. However, the manual/paper-based normal distribution curve before the pandemic and the normal curve shifting to the right with mobile apps indicate an increase in the mean score (Fig. 2). Furthermore, changes in the pandemic period, i.e., before, during, and after the pandemic, did not affect the results of rank-based peer assessment carried out on a paper-based basis and mobile apps.

Rank-based peer assessment can be implemented in educational settings to foster a more collaborative and reflective learning environment. Research conducted by Concina [19] demonstrated that incorporating rank-based peer assessment into the evaluation process resulted in improved student engagement, critical thinking, and self-regulated learning. When students were given the opportunity to rank their peers’ work based on predefined criteria, they were more actively involved in the learning process and developed a deeper understanding of the subject matter. Additionally, the use of rank-based peer assessment encouraged students to provide constructive feedback to their peers, promoting a sense of responsibility and accountability within learning communities. Performance metrics arranged based on specific questions in assessing student performance including timeliness in attending each group activity, responsibility and accountability in completing agreed-upon group tasks and field visits, effective communication with all group members, ability to coordinate and collaborate with group members, ability to respect for group members’ opinions, portion of contribution to program implementation according to the plan, and communication with partner families and the community. Furthermore, Song et al. [20] showed rank-based peer assessment exhibit a minimum of 10% higher reliability compared to the evaluators in rating-oriented peer assessment. Additionally, a detailed examination revealed that the evaluators in ranking-based assessments show a tendency to accurately evaluate artifacts that are more distinct from one another, whereas no such trend was observed among rating-based evaluators.

Student mean scores increased significantly during the pandemic period, which coincided with the use of mobile apps and lasted until after the pandemic (Fig. 3). So, rank-based peer assessment is proven to produce normally distributed scores. Scores normally distributed are more objective where rank-based peer assessment can overcome the problem so far, namely failure to fail which is students tend to score the same as the best score [17].

Furthermore, the normal curve that shifts to the right shows that the mean scores are higher than before (Figs. 2 and 3). This could happen for a number of possible causes. First, during the pandemic, students stayed at home and were focused and serious about running the CFHC-IPE program online and also focused on online peer-assessing. Second, the curve shifting to the right when using webapps assessment methods may be due to the difference in the value range of assessment. The rating range against manual assessment was 1–5, whereas rating range against webapps method of assessment was 1–10. One of the characteristics of peer assessment is the occurrence of failure to fail [19]. The tendency of students to assess their friends with good grades was observed. In this study, we argue that the failure-to-fail might occur due to a number of reasons. Firstly, students may feel pressure to maintain positive relationships with their peers and may worry that being too critical in their assessments could damage these relationships. Additionally, students may be more inclined to give their friends the benefit of the doubt or to overlook mistakes or weaknesses in their work. It is a potential for bias, specifically, due to a tendency for students to be overly generous when evaluating the work of their friends, giving them higher grades than they might otherwise deserve [19].

Moreover, the insignificant increase in the average score between the pandemic and post-pandemic periods may indicate that the pattern of rank-based peer assessment by students has remained the same, even though the pandemic has ended. Rank-based peer assessment which are facilitated with mobile-apps do have the advantage of being able to assess objectively, accessible from anywhere and at any time which is suitable for implementation in IPE programs that involve many community-based people and students are far apart from each other. As shown in Figs. 2 and 3, rank-based peer assessment presented an objective assessment. The objectivity of the assessment method is demonstrated by the normal distribution curve of the result of assessment regardless of the methods used, both manual and webapps methods. Objectivity is a vital requirement when it comes to assessment in any field. It refers to the ability to evaluate and judge something fairly, impartially, and without any personal bias or prejudice. In educational settings, objectivity in assessment ensures that students are evaluated based on their performance and knowledge rather than any other extraneous factors. Assessment results must be reliable, consistent, and unbiased to accurately measure a student’s academic progress and achievement [21]. Objectivity is particularly important which can significantly impact a student’s future performance and competencies achievement.

The use of objective assessment measures helps to ensure fairness and equity in the evaluation process, which is especially important in diverse classrooms where cultural and socio-economic factors can influence academic achievement. Objective assessments help to ensure that students from all backgrounds have an equal opportunity to demonstrate their knowledge and skills [21]. Therefore, rank-based peer assessment that has been used to assess this activity can be implemented as an alternative method of assessment of interprofessional education activity, thus helping the involvement of diverse professions of students.

The development of mobile apps undoubtedly demands a variety of resources. As suggested by an integrative literature review [22], financial and infrastructure are among the challenges and barriers of maintaining educational technologies in health professions education. In the industrial era 5.0, technology was always developed to support learning and assessment as is seen in this study.

There is a possibility of peer grading bias, which might lead to inaccurate assessments. This is where students might receive higher or lower scores than what they deserved, and the possibility of ‘free-loaders’ [23]. The phenomenon might impact the overall fairness and validity of the assessment process, including the credibility of the educational institution. The use of clear and objective assessment criteria and peer assessment training are pivotal to mitigate the potential derailment. Moreover, faculty should consider strategies such as anonymous peer assessments, using double-blinded approach to help minimize bias [19].

Despite curve shifts to the right, the scores seen in our study are still within normal distribution. Hence, helping to distinguish between high and low performing students. Our experience in using mobile application for interprofessional peer-assessment has encouraged it to become a preferable approach during the pandemic. The nature of mobile applications is interesting and easily accessible by learners and flexible [24]. In addition, the mobile application has made facilitating assessment and reflection possible as a distance learning modality [25], in remote communities and rural areas. It is important to note the possible drawbacks on the use of online applications for learning. For instance, applications might need a good internet connection to operate optimally, which can be difficult in certain rural areas with bandwidth limitations [22]. In addition, facilitators’ supervision might be challenged due to a lack of face-to-face interaction [26].

The findings from this study may be generalizable to similar populations of undergraduate health science students in comparable academic structures, particularly within interprofessional education programs in healthcare education fields. However, the extent to which these findings apply to students in non-health disciplines or in regions outside of Southeast Asia may be limited due to contextual and cultural differences. Further studies would be valuable to confirm these findings in diverse geographical and educational settings to strengthen the broader applicability of the results.

### Limitations

This study might have a limitation on the scoring differences between years/batches as the assessment tools are still under development. However, we standardized the assessment scores to the 0-100 range to ensure comparability [27]. The study focused on the peer assessment aspect of the program, and requires confirmation using other assessment results.

## Conclusion

The use of rank-based peer assessment using mobile application for a longitudinal interprofessional education is possible for an objective and safe peer assessment and learning facilitation during and after pandemic. This study shows that the use of mobile applications does not change the distribution of rank-based score, but resulted on better mean score.

Future study on the impact of the peer assessment on the students’ interprofessional practice during the clinical learning phase should be undertaken to evaluate the long-term impact of the intervention. It is recommended that the mobile application should also be expanded for other learning activities. The peer assessment results may also be compared with other assessment items to ensure a comprehensive 360-degree assessment and summarized into portfolio.

## Electronic supplementary material

Below is the link to the electronic supplementary material.

Supplementary Material 1

Supplementary Material 2

Supplementary Material 3

Supplementary Material 4

Supplementary Material 5

Supplementary Material 6

Supplementary Material 7

## Data Availability

https://drive.google.com/drive/u/0/folders/1Idt4olPrg5SkTCo4MLpKNth_UgJM1O2c.
