# Supplementary figures and images for: Comparing paper-based and mobile application for rank-based peer assessment in interprofessional education: before, during, and after the COVID-19 pandemic
Source: BMC Med Educ. 2024 Nov 27;24:1383. doi: 10.1186/s12909-024-06382-2 (PMC11600818; doi:10.1186/s12909-024-06382-2)

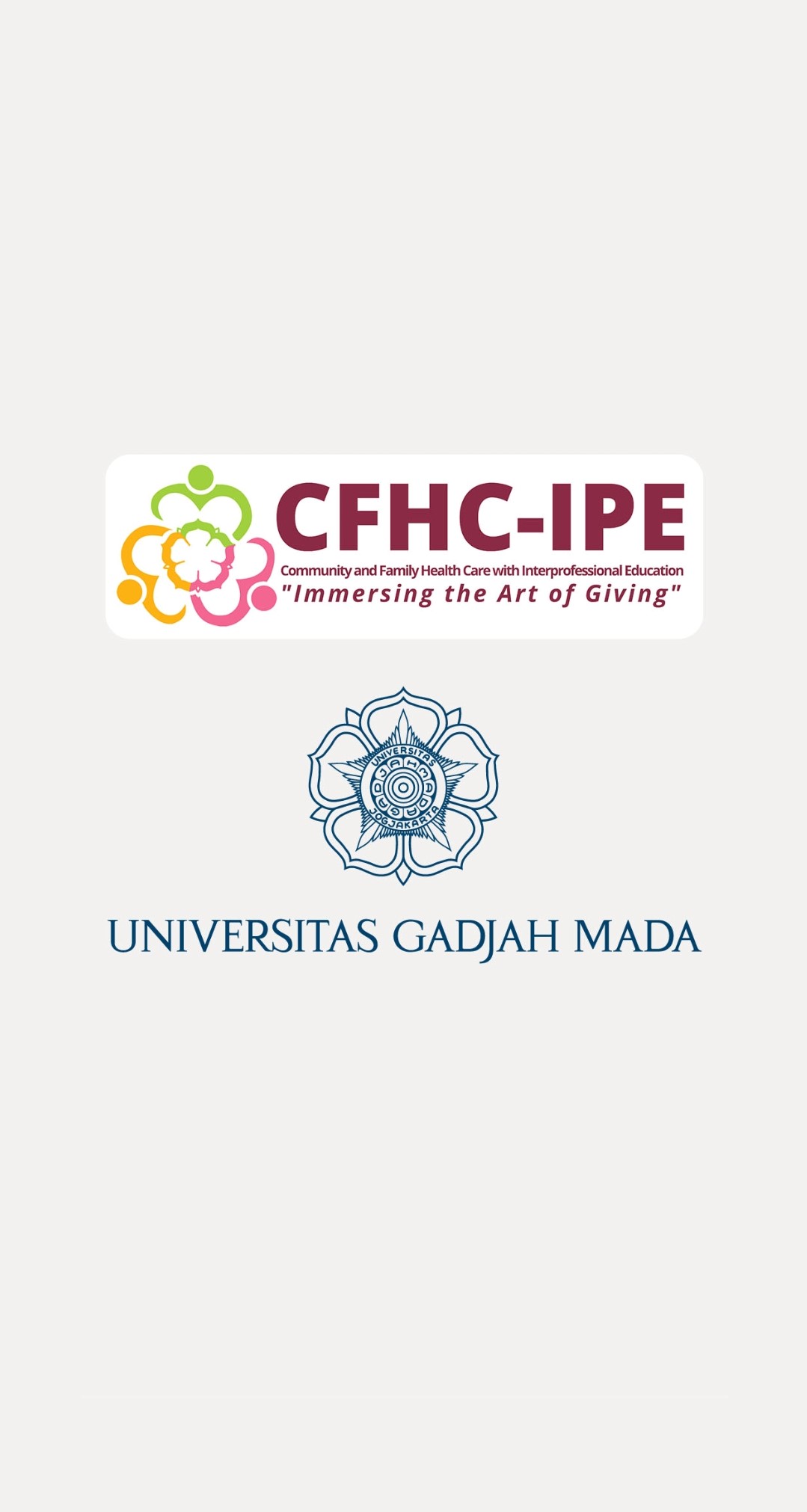

Supplement: Supplementary file 1 — Supplementary Material 1 [file 12909_2024_6382_MOESM1_ESM.jpg]

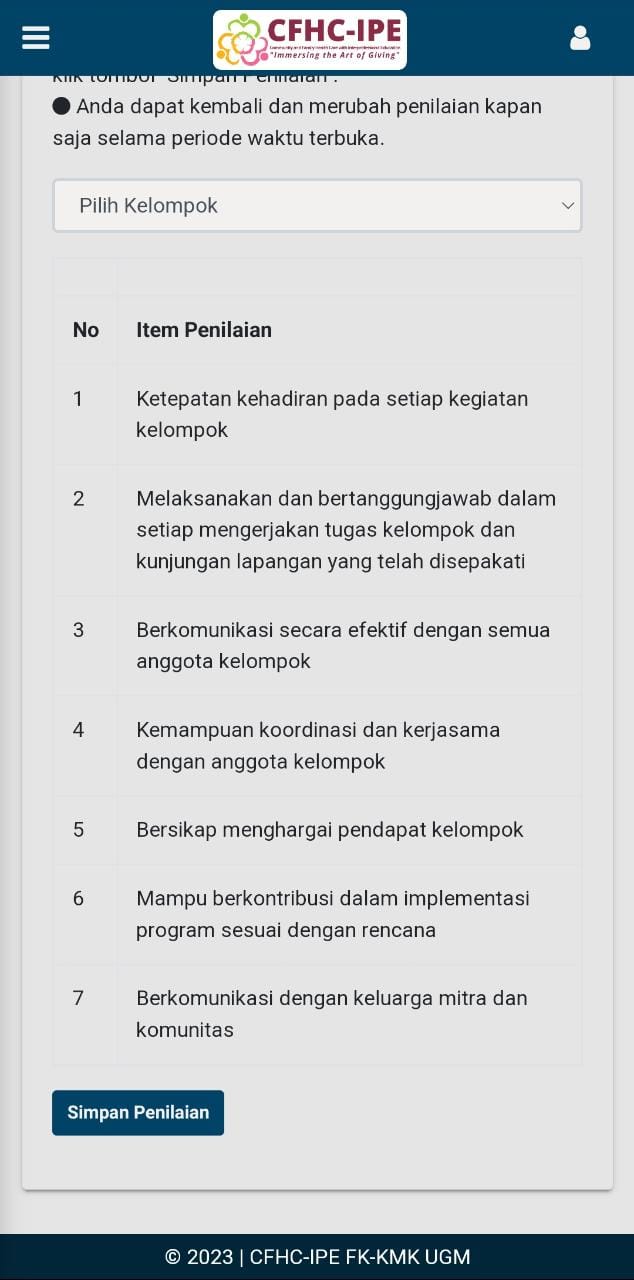

Supplement: Supplementary file 2 — Supplementary Material 2 [file 12909_2024_6382_MOESM2_ESM.jpg]

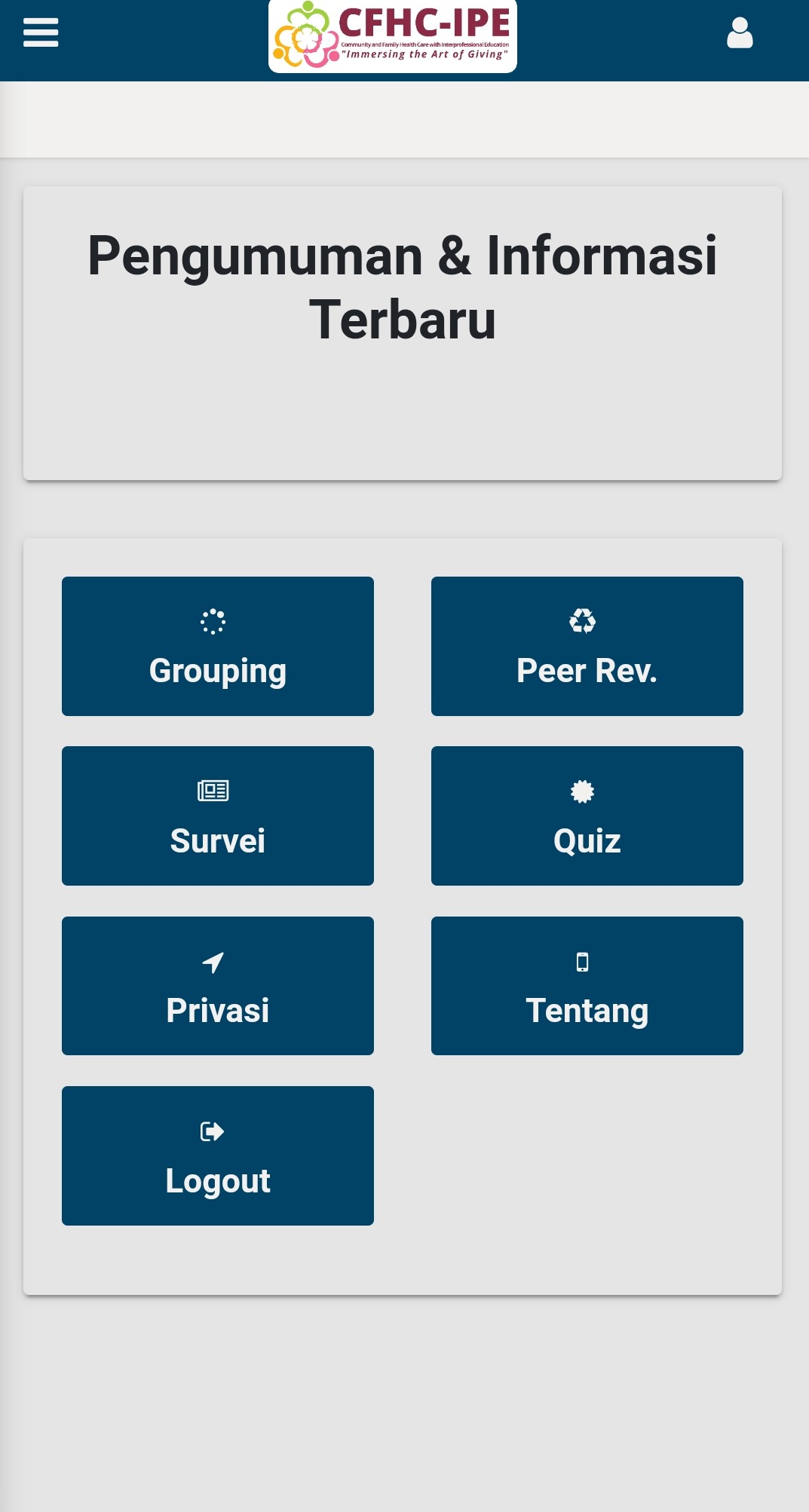

Supplement: Supplementary file 3 — Supplementary Material 3 [file 12909_2024_6382_MOESM3_ESM.jpg]

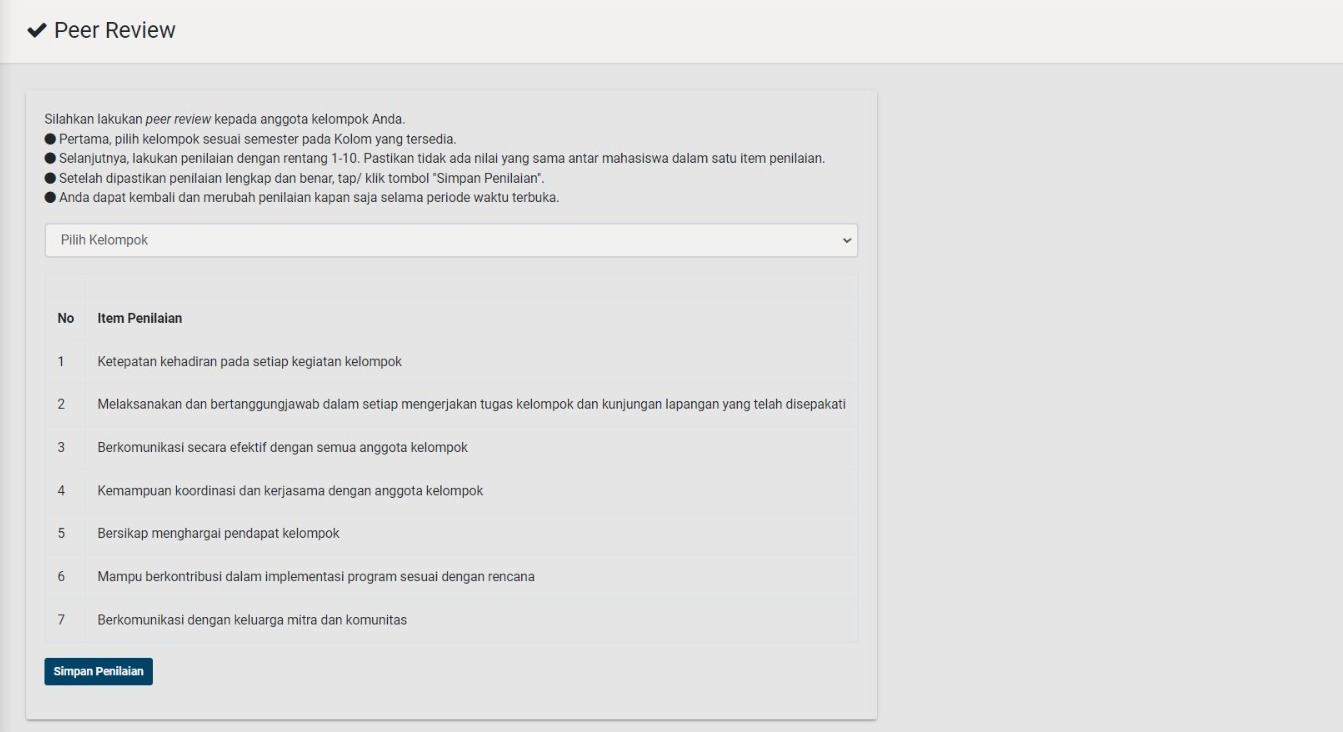

Supplement: Supplementary file 4 — Supplementary Material 4 [file 12909_2024_6382_MOESM4_ESM.jpg]

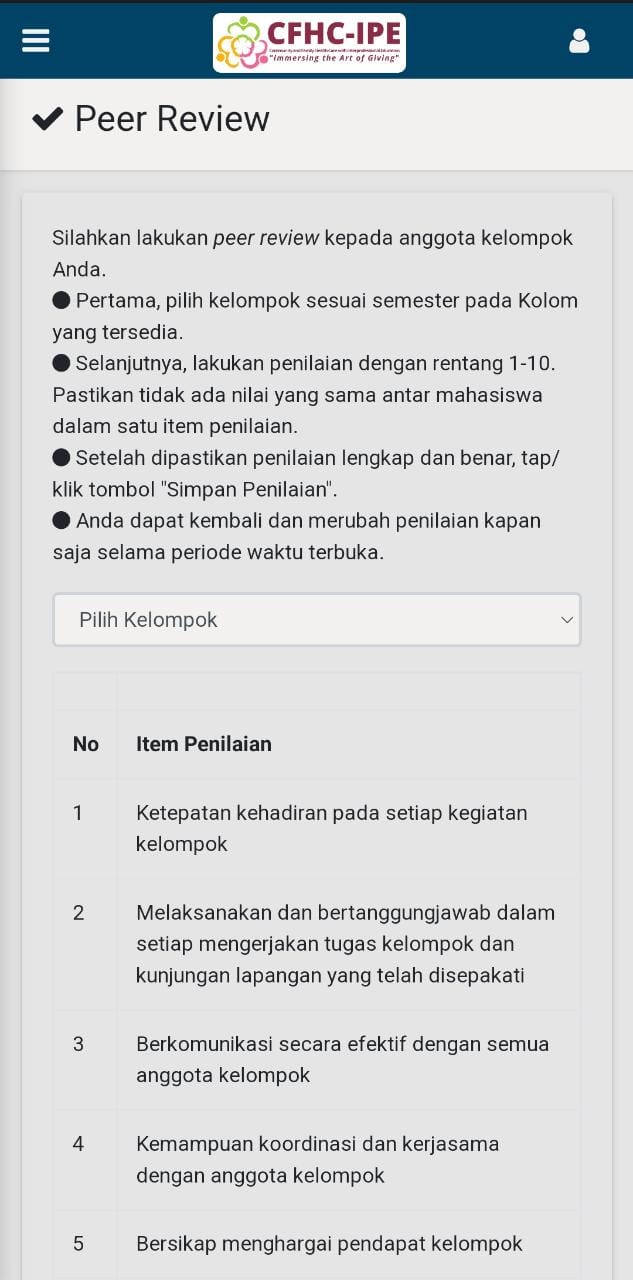

Supplement: Supplementary file 5 — Supplementary Material 5 [file 12909_2024_6382_MOESM5_ESM.jpg]

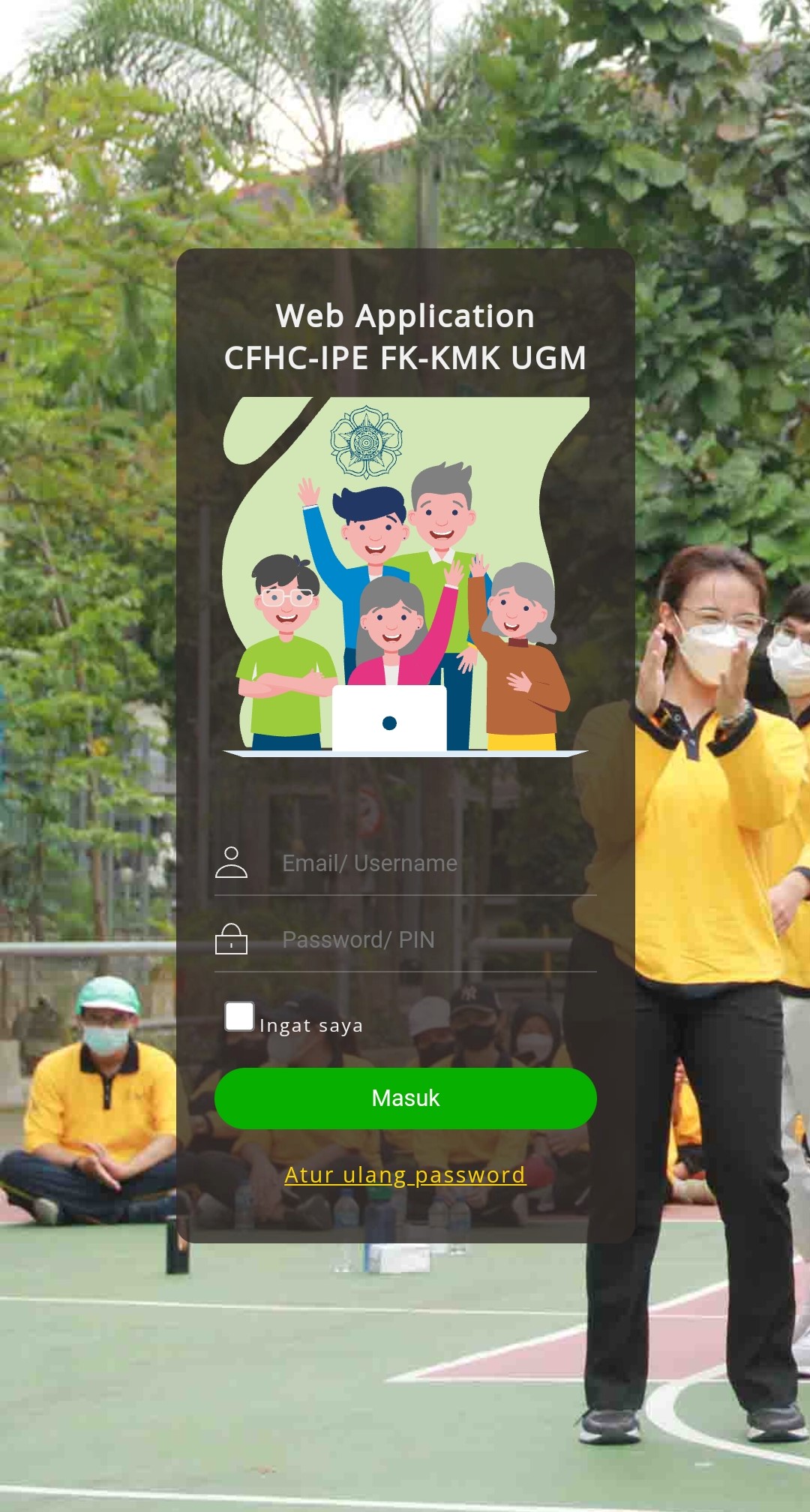

Supplement: Supplementary file 6 — Supplementary Material 6 [file 12909_2024_6382_MOESM6_ESM.jpg]
